# Early Loss of Telomerase Action in Yeast Creates a Dependence on the DNA Damage Response Adaptor Proteins

**DOI:** 10.1128/MCB.00943-15

**Published:** 2016-06-29

**Authors:** Kyle A. Jay, Dana L. Smith, Elizabeth H. Blackburn

**Affiliations:** a W. M. Keck Science Department, Claremont McKenna College, Claremont, California, USA; b Department of Biochemistry and Biophysics, University of California, San Francisco, California, USA

## Abstract

Telomeres cap the ends of chromosomes, protecting them from degradation and inappropriate DNA repair processes that can lead to genomic instability. A short telomere elicits increased telomerase action on itself that replenishes telomere length, thereby stabilizing the telomere. In the prolonged absence of telomerase activity in dividing cells, telomeres eventually become critically short, inducing a permanent cell cycle arrest (senescence). We recently showed that even early after telomerase inactivation (ETI), yeast cells have accelerated mother cell aging and mildly perturbed cell cycles. Here, we show that the complete disruption of DNA damage response (DDR) adaptor proteins in ETI cells causes severe growth defects. This synthetic-lethality phenotype was as pronounced as that caused by extensive DNA damage in wild-type cells but showed genetic dependencies distinct from such damage and was completely alleviated by SML1 deletion, which increases deoxynucleoside triphosphate (dNTP) pools. Our results indicated that these deleterious effects in ETI cells cannot be accounted for solely by the slow erosion of telomeres due to incomplete replication that leads to senescence. We propose that normally occurring telomeric DNA replication stress is resolved by telomerase activity and the DDR in two parallel pathways and that deletion of Sml1 prevents this stress.

## INTRODUCTION

Telomeres are composed of repetitive DNA sequences and their bound protective proteins at the ends of linear eukaryotic chromosomes. These repetitive sequences buffer against the loss of terminal sequences due to incomplete DNA replication and, through sequence-specific binding of protein factors, distinguish the chromosome end from a potentially dangerous DNA double-strand break (DSB). Saccharomyces cerevisiae cells constitutively express telomerase, which lengthens telomeres and allows bulk populations to grow indefinitely. Following a mutation inactivating telomerase, yeast cells can continue cycling for approximately 60 to 80 divisions before their telomeres become critically short and can no longer maintain the protective complement of proteins. At this point, here referred to as late after telomerase inactivation (LTI), the telomere becomes deprotected and activates a Mec1-dependent DNA damage response (DDR), leading to a permanent cell cycle arrest known as senescence. Here, we studied the phenotypes of cells early after telomerase inactivation (ETI), while telomeres were still long, in order to determine the conditions under which telomerase activity was required for normal viability.

Telomeres are increasingly recognized as genomic regions prone to replication stress and impaired DNA replication fork movement in baker's yeast (1), fission yeast (2), and mammals (3–6). In addition, many of these studies have found that telomere binding proteins (2, 5–8) or telomerase activity (1, 4) is required to suppress or survive this replication stress or that stalled replication forks may act as telomerase substrates (9). This is consistent with the discovery of several interactions between telomerase or telomere components and the lagging-strand DNA replication machinery (8, 10, 11), suggesting that proper replication of the telomere requires the coordinated actions of many telomere-associated factors. In addition to being highly repetitive and tightly bound by protein, telomeric DNA sequences are also very G rich, and single-stranded telomeric DNA forms highly stable G-quadruplex structures that inhibit DNA replication in mammalian systems (12). Overall, telomeres present a difficult landscape for the DNA replication machinery.

Telomere deprotection resulting from critically short telomeres shares many properties with classic DNA damage (13–15). In addition, many classic DDR proteins bind telomeres and have functions in telomere maintenance (16). DNA damage signaling in budding yeast is primarily initiated by two upstream phosphatidylinositol 3-kinase (PI3K)-related kinases, Mec1 and Tel1 (17). In addition to their functions in the DDR, in telomerase wild-type (WT) cells, Mec1 and Tel1 have a slight and a major role, respectively, in telomere length regulation, and cells lacking both kinases senesce as if they did not have active telomerase (18).

The Mec1 and Tel1 proteins are functionally redundant to some degree but are also able to respond to distinct types of DNA damage. For example, Mec1 is required to sense the single-stranded DNA that arises in response to replication stress and stalled DNA replication forks (19). Deletion of Mec1 is lethal but can be suppressed by deletion of Sml1, which results in elevated deoxynucleoside triphosphate (dNTP) pools and facilitates DNA replication (20, 21). Tel1 is thought to be more important for the detection, processing, and repair of DNA double-strand breaks (22) and has been shown to be protective against telomeric end-to-end fusions (23).

Downstream of Mec1/Tel1 in the yeast DDR are two semiredundant adaptor proteins, Mrc1 and Rad9. Mrc1 is required for the DNA replication stress response and travels as a component of the DNA replication fork (24, 25). In addition, Mrc1 becomes activated in response to the extensive telomere erosion of LTI cells and has been shown to protect uncapped telomeres from exonuclease activity (26, 27). Rad9 is also important for the response to DNA damage, as cells lacking Rad9 are especially sensitive to DSB-inducing chemicals and UV/ionizing radiation (28, 29). In response to DNA damage, one or both of these adapter proteins becomes phosphorylated and mediates signaling to downstream kinases and target proteins (24, 30). Through this signaling cascade, a multitude of actions can be taken in response to the recognition of DNA damage, which will eventually result in the resolution of the genetic insult in question and the maintenance of genomic stability. Here, we present phenotypes showing that, unlike in WT cells, ETI mutations induce dependence on functional DNA damage response adaptor proteins for viability, despite the absence of any exogenously introduced DNA damage.

We examined two settings in which there was a lack of telomerase action on telomeres, chosen because they are situations that cause no gross changes in the growth of the yeast. The first setting was in ETI cells examined well before there were any signs of senescence. The telomerase enzyme can be inactivated in a number of ways. Here, telomerase activity was eliminated either by deletion of the template-containing RNA component (*tlc1*Δ), by deletion of the catalytic reverse transcriptase subunit (*est2*Δ), or by replacing the reverse transcriptase subunit with a catalytically dead allele (*est2D530A*) (31). This catalytically dead allele is mutated at an invariant aspartate residue, which is one of three aspartate residues required for phosphoryl transfer in reverse transcriptases (32). The two deletion mutations result in disassembly of the telomerase complex, whereas the catalytically dead point mutant results in an intact telomerase complex that is unable to extend the 3′ G-rich telomeric overhang, as it lacks DNA polymerization activity. All three mutants cause bulk telomeres to shorten at the same rate. Heterozygous diploids containing a single copy of these telomerase mutations were sporulated, and haploid ETI cells were freshly isolated from the meiotic products. These haploid ETI cells still grew indistinguishably from WT control cells, but the telomeres were beginning to shorten. The second setting was cells constructed to have preelongated (longer than WT) telomeres, followed by removal of the gene construct causing the telomere hyperelongation. It has been shown that telomerase acts only on shorter telomeres, and thus, it is presumed to act little if at all on such experimentally greatly elongated telomeres. In these cells, telomeres were also shortening despite the presence of telomerase, a passive process that takes place over several tens of cell generations before telomere lengths eventually shorten back to WT lengths (33, 34).

We report that, well before senescence in both these settings lacking telomerase action on telomeres, the combined absence of the functions of the DNA damage adaptor proteins Mrc1 and Rad9 induces lethality. Notably, in the pre-elongated-telomere setting, this lethality occurred even though the telomerase was WT. The phenotypes we report are indicative of a response to the lack of telomerase action on telomeres that engages specific DNA damage checkpoint signaling components.

The findings presented here expand on and further support a model we recently presented (35) in which dependence on DDR components in ETI cells results from an inability to resolve DNA replication stress in the telomere if both DDR and telomerase function are absent. Elevation of dNTP pools in the cell, known to facilitate DNA replication (36), alleviates this dependence to near-WT levels. Therefore, it has become apparent that in S. cerevisiae, even independent of bulk telomere length, an active telomerase enzyme is a requirement to ensure telomere replication, a requirement that can be alleviated by facilitating DNA replication. Furthermore, lack of telomerase action on telomeres becomes lethal specifically in the setting of loss of DNA damage pathway adaptor protein function.

## MATERIALS AND METHODS

### Yeast strain construction.

Plasmid and oligonucleotide sequences are available upon request. The strain background was W303 or S288C (and isogenic with BY4736) (23), as indicated. Diploids were isolated on selective medium by double auxotrophy selection with subsequent sporulation on solid plates (W303) or by visual isolation of zygotes after 5 h of mating and liquid sporulation (S288C). Complete disruption of open reading frames (ORFs) was carried out by using PCR-mediated gene disruption (37). Disruption cassettes for *NAT* or *HYGMX4* were as described previously (38). Galactose promoter regulation was performed using PCR-mediated insertion of the promoter upstream of the desired coding sequence as described previously (39). RNR1 overexpression was accomplished by PCR-mediated insertion of the pTEF promoter upstream of the RNR1 coding sequence. Overexpression was verified via quantitative reverse transcription (RT)-PCR. Altered telomerase genes were covered with pRS CEN/ARS plasmids containing either the wild-type *TLC1* or *EST2* with endogenous promoters and terminators and were sequence verified.

All *mrc1*^*AQ*^ strains in the 3,000 series were made by first deleting MRC1, and then pRS403-*mrc1*^*AQ*^ was digested with BspEI and integrated into the *mrc1*Δ strain at the endogenous *MRC1* promoter. The *MRC1* gene, including 600- and 400-bp upstream and downstream regions, respectively, was PCR amplified and cloned into pRS403 using SalI and BamHI cloning sites to yield pEHB3201. pEHB3201 was used to construct pEHB3202, a plasmid bearing the *mrc1*^*AQ*^ allele (40), cloned into pRS403. SQ and TQ motifs on the N terminus of the gene were mutated to AQ by assembly of an ∼900-bp gene fragment from oligonucleotides and cloning of the fragment into pEHB3201 digested with SacI and EcoRI. SQ and TQ motifs in the central and C-terminal portions of the gene were mutated to AQ using an overlap extension PCR strategy (38). All *mrc1*^*AQ*^ strains in the 30,000 series were made by cloning the *mrc1*^*AQ*^-containing SalI-BamHI fragment of pRS403-*mrc1*^*AQ*^ into pRS406. pRS406-*mrc1*^*AQ*^ was digested with BspEI and integrated into the WT strain 3056. 5-Fluoroorotic acid (5-FOA) selection was used to select for loop-outs, and strains in which *MRC1* was fully replaced by *mrc1*^*AQ*^ were identified by PCR analysis.

### Growth of mutants for monitoring early loss of telomerase.

ETI cells are defined as cells without telomerase for approximately 25 to 30 generations and were produced by two methods: sporulation of diploid heterozygote strains (*tlc1*Δ/*TLC1* or *est2D530A*/*EST2*) or by loss of a covering plasmid in a haploid telomerase-deficient background strain streaked on solid medium. Diploid strains were passaged 3 to 5 times (depending on the genotype) to equilibrate telomere lengths before sporulation. Fully grown spore colonies (2 days of growth at 30°C) contained cells that had been without telomerase for approximately 20 generations. Cells derived from these original spore colonies, or telomerase-negative colonies after plasmid loss, were passaged (10 to 20 additional generations via patching, streaking, or growth in liquid culture) and are referred to as passage 1 cells.

Because populations of cells lacking telomerase action on telomeres eventually undergo senescence after 50 to 80 cell divisions, when the telomeres have become critically shortened, we performed most of our analyses at 25 to 30 generations after genetic removal of active telomerase, when the telomeres were still relatively long and well before senescence of the bulk cell population. We confirmed that these ETI cells showed healthy growth by the following criteria: cell and colony morphologies were wild type; 95% of the cells entered a budding cycle following release from α-factor arrest and continued growing through at least the next full cell cycle; and there was no detectable Rad53 or Mrc1 phosphorylation, which normally becomes detectable only later after telomerase loss, at the onset of senescence of the cell population

### Southern blotting analysis of telomere length.

Genomic DNA was prepared from cells collected from the densest portions of serial streaks on solid medium after the indicated number of passages. Genomic DNA was then digested with XhoI and run on 0.8% agarose gels alongside an NEB 1-kb DNA ladder. The DNA was transferred from the gels to Hybond N+ membranes and probed with a γ-^32^P-end-labeled WT telomeric-repeat oligonucleotide (5′-TGTGGTGTGTGGGTGTGGTGT-3′), as described previously (37, 38). The membranes were exposed using Amersham Biosciences storage phosphor screens and visualized using a Typhoon 9400 variable-mode imager.

### PCR assay for deprotected telomeres.

A PCR assay for deprotected telomeres was performed as described previously (23) with slight modifications. Diploid strains that were heterozygous for all mutations, expressed an HO endonuclease gene driven by a galactose-inducible promoter, and contained a modified chromosome VII-L containing an HO cut site were created. Colonies of the desired genotypes were taken directly from dissection plates and grown overnight in 2.5% raffinose medium. The colonies were then induced for 8 h in 3% galactose. Cells were pelleted, and genomic DNA was purified using a glass bead and phenol chloroform DNA preparation. PCR mixtures consisted of 5 μl Qiagen Q solution, 2.5 μl Qiagen 10× PCR buffer, 0.5 μl dNTPs (10 mM each), 0.125 μl (100 μM) of each primer (5′-CGCGCGCGGCCGTGACATGGTTATAACTGTTAGC-3′ and 5′-GTCAGTGATTATGTATTGTGTAGTATAGTATATTGTAAG-3′), 0.5 μl of Platinum *Taq*, 11.25 μl water, and 100 ng genomic DNA in 5 μl water (25-μl total volume). The following cycling conditions were used: 95°C (5 min); 35 cycles of 95°C (5 s), 52°C (30 s), and 72°C (1 min); and 72°C (5 min).

### Serial streaking and serial dilution assays.

Serial streaks were made using cells from original dissection colonies after genotyping. They were passaged on solid yeast extract-peptone-dextrose (YPD) medium and underwent 2 days of growth at 30°C before imaging. Multiple individual colonies from a given streak/passage were used to create the next streak in the series. Each streak represented approximately 20 cell divisions. For serial-dilution experiments, genotyped dissection colonies were grown overnight in liquid culture (5 to 10 divisions) and diluted to an optical density (OD) (*A*_600_) of ∼0.1 to 0.2. The cells underwent approximately 4 h of growth at 30°C and were placed on ice. The cells were sonicated, and the cell concentration was measured using a hemocytometer. Cells (4 × 10^5^) were resuspended in 100 μl of appropriate medium, and 5-fold serial dilutions were plated on yeast extract-peptone (YEP) agar medium containing either 2% dextrose or galactose and the indicated drugs (if applicable) at the specified concentrations.

### Cell cycle analysis.

Cultures were inoculated from plates and grown overnight. At an OD (*A*_600_) of ∼0.2, 0.5-ml samples were fixed as described above for cell cycle analysis. α-Factor was added to a final concentration of 5 μg/ml. One hour after the addition of α-factor, the culture was split into two equal parts. After 2 h and 20 min of α-factor arrest, samples were fixed for the zero time point (*t* = 0) (with respect to α-factor). After 2 h and 30 min, the cells were harvested by filtration and washed twice with 50 ml of YPD medium prewarmed to 30°C in order to remove residual α-factor. The samples were fixed in ethanol every 15 min for 120 min after release from α-factor.

Cells were analyzed by fluorescence-activated cell sorting (FACS). Ethanol-fixed cells were pelleted, washed twice in 50 mM sodium citrate, pH 7.5, and resuspended in 600 μl of 50 mM sodium citrate, pH 7.5. Two hundred microliters of 1-mg/ml RNase A (Qiagen) was added, and samples were incubated in a 50°C water bath for 1 h; 40 μl of 20-mg/ml proteinase K (Roche) was added, and the samples were incubated an additional 1 h at 50°C. Volumes for all the preceding steps were scaled down for asynchronous times points, as they usually contained fewer cells. After proteinase K treatment, 250 to 300 μl of cells was diluted to a final volume of 1 ml in 50 mM sodium citrate, pH 7.5/SYBR green I (Molecular Probes). SYBR green I was present at a final dilution of 1:500 of the commercial stock. Samples were left at 4°C overnight in the dark. Triton X-100 was added to a final concentration of 0.25%. The samples were sonicated using a Branson 450 Sonifier equipped with a double-step tip at an output setting of 2 and a duty cycle of 30% for 3 or 4 pulses. Finally, the samples were filtered, and data were acquired on a FACSCalibur (BD BioSciences).

## RESULTS

### Early telomerase inactivation imposes dependence on a functional DNA damage response for viability.

**FIG 1:**
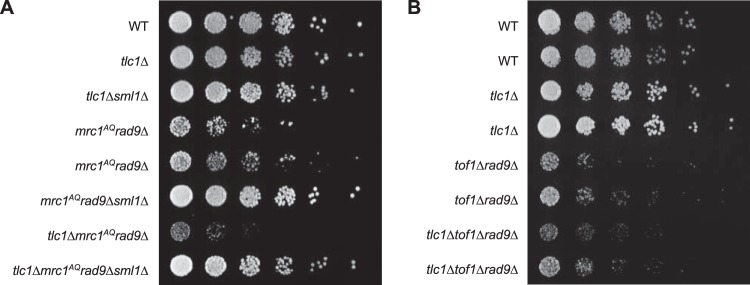
Epistasis analysis of Tel1 and Mrc1 mutants on G_2_/M cell cycle extension and cell viability. (A) ETI *mrc1*^*AQ*^ *rad9*Δ synthetic phenotype shown via semiquantitative serial dilutions. ETI cells show reduced colony formation and colony size in the absence of functional Mrc1 and Rad9 proteins. Colony size and growth phenotypes are restored with deletion of Sml1. (B) Deletion of the Mrc1 partner protein, Tof1, does not mimic the ETI synthetic phenotype. Two biological replicates are shown.

**FIG 2:**
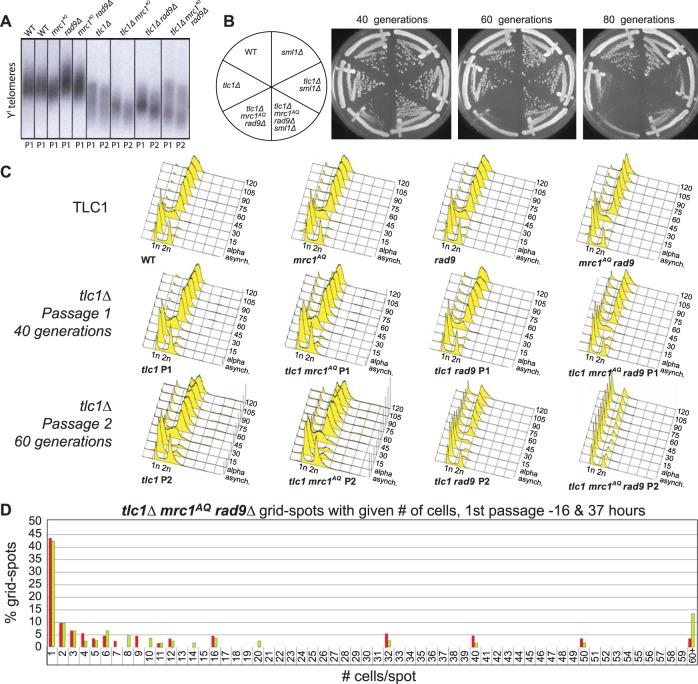
ETI, combined with loss of MRC1 and RAD9, leads to massive cell death without excessive telomere shortening. (A) Southern blot, using a Y′ telomere-specific probe to measure telomere length. (B) Serial streaks of freshly sporulated *tlc1*Δ strains starting approximately 20 generations after telomerase loss. The first, second, and third streaks represent cells after 40, 60, and 80 generations of telomerase loss, respectively. (C) FACS analysis of cells prepared from plates after serial streaks. Cultures were grown up, and samples were collected every 15 min following α-factor arrest and release. (D) WT and *tlc1Δ mrc1*^*AQ*^ *rad9* strains were freshly sporulated and dissected on YPD plates. Serial streaks were made from spore colonies, and 100 unbudded cells were identified by microscopic visualization and gridded individually onto YPD plates. The numbers of cells in descendant colonies were determined for each gridded cell after 16 and 37 h for the first two streaks (passage 1 is shown). Most cells ceased dividing as unbudded cells. Comparable wild-type cells yielded colonies so dense that cell numbers could not be determined. Red bars represent the first passage at 16 h, and green bars represent the second passage at 37 h.

**FIG 3:**
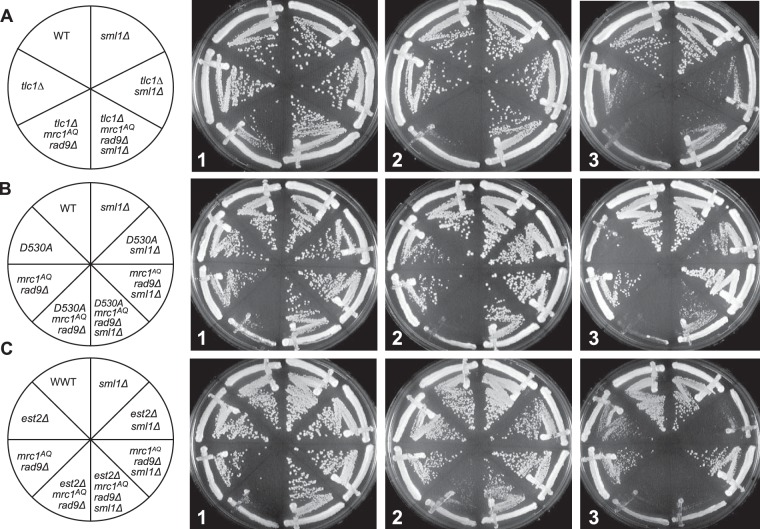
ETI mutants show dependence on the DNA damage response that can be alleviated by Sml1 deletion. Three different ETI mutants, the *tlc1*Δ (A), *est2D530A* (B), and *est2*Δ (C) mutants, showed loss of viability in strains lacking a functional DNA damage response due to Mrc1 and Rad9 mutation. Deletion of the RNR inhibitor, Sml1, alleviated this requirement (compare left and right halves of the plates). First-passage cells were taken directly from sporulation of diploid strains heterozygous for all the mutations. Three passages are shown (left to right), each representing approximately 20 generations.

We previously reported that individual ETI yeast mother cells displayed a reduction in mother cell life span and greater heterogeneity of cell cycle durations than the WT (35). Mutations in one half of the DDR (*mrc1*^*AQ*^ or *tel1*Δ) exacerbated the cell cycle and accelerated mother cell aging phenotypes observed in ETI cells, while mutations in the other half of the pathway (*mec1Δ sml1*Δ and *rad9*Δ) had little to no effect. To further test the involvement of the DDR in the phenotypes of ETI cells, we inactivated both halves of the DDR, completely eliminating the cell's ability to respond to DNA damage. We simultaneously inactivated the semiredundant adaptor proteins Mrc1 and Rad9, which together constitute the second tier of the DDR response. Without Mrc1 and Rad9 function, the upstream PIKK kinases are unable to phosphorylate their downstream targets, effectively negating the DDR (17, 24, 30). For these experiments, we used the *mrc1*^*AQ*^ allele, which retains its function as a component of the replication fork but has all 17 possible PIKK phosphorylation sites mutated by serine-to-alanine substitutions (40). ETI cells lacking Mrc1 and Rad9 function (ETI *mrc1*^*AQ*^ *rad9*Δ) showed a drastic drop in viability and colony growth at the bulk population level for all three ETI mutations tested (Fig. 1A, 2, and 3). FACS and pedigree analysis showed that the ETI *mrc1*^*AQ*^ *rad9*Δ cells stopped growing as small, unbudded cells with G_1_ DNA content, possibly due to mitotic catastrophe (Fig. 2C and D). Therefore, ETI strains become nonviable in the complete absence of a functional DNA damage response. Strikingly, as with our ETI yeast mother cell experiments, the deletion of *SML1* completely rescued synthetic lethality for all ETI *mrc1*^*AQ*^ *rad9*Δ strains. The colony formation and growth defects were completely suppressed, and these cells grew similarly to the WT (Fig. 1A and 3).

### The *mrc1*^*AQ*^ *rad9*-induced synthetic lethality with ETI is specific to Mrc1 activity.

The Mrc1 protein not only facilitates signaling from the upstream PIKK kinases but also travels as part of the replication fork in complex with two other proteins, Tof1 and Csm3, which are required for Mrc1 association with the fork (25). In order to determine if the *mrc1*^*AQ*^ *rad9*Δ-induced synthetic lethality in ETI cells was specific to Mrc1 activity, as opposed to Mrc1 association at replication forks, we performed an experiment in which Tof1 was deleted in place of mutating Mrc1. In the *tof1*Δ *rad9*Δ and ETI *tlc1*Δ *tof1Δ rad9*Δ strains, there appeared to be no additional effect from the ETI mutation (Fig. 1B). Therefore, loss of association of Mrc1 with the replication fork is not sufficient to induce synthetic lethality in combination with ETI and Rad9 deletion mutations. Rather, our results indicate that Mrc1 signaling activity must be eliminated to fully disable the DDR and induce the synthetic phenotype seen in ETI *tlc1Δ mrc1*^*AQ*^ *rad9*Δ cells.

**FIG 4:**
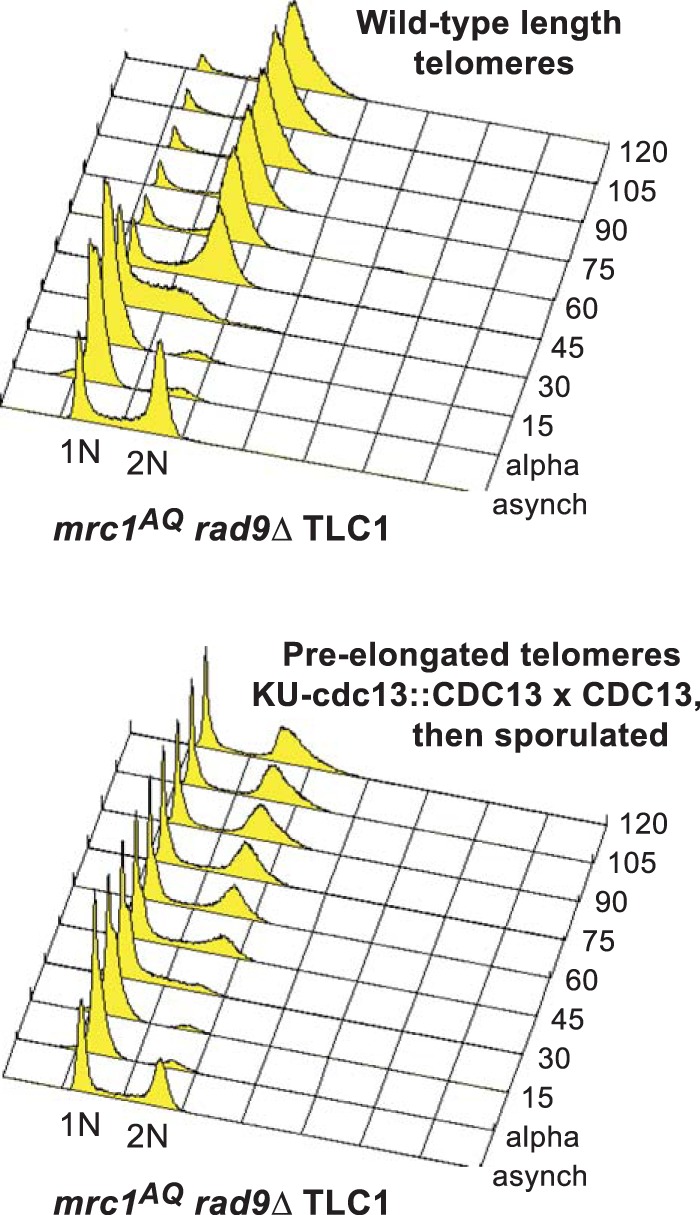
*mrc1*^*AQ*^ *rad9* cells arrest in G_1_ when telomeres lack telomerase. (B) In yEHB3284, a heterozygous diploid strain, telomeres were elongated through expression of Cdc13-Ku70p. After sporulation, *mrc1*^*AQ*^ *rad9* CDC13 cells with preelongated telomeres were selected and synchronized through α-factor arrest and release. Samples were collected before synchronization and every 15 min thereafter and analyzed by FACS.

We wished to determine the mechanism behind the *sml1*Δ rescue of the ETI-DDR synthetic lethality. We showed previously that *sml1*Δ does not lead to telomere elongation (35). To further test whether these phenotypes were simply a consequence of short telomeres, we constructed cells that had their telomeres preelongated through expression of a CDC13-Ku70 fusion under the CDC13 promoter, which tethers excess telomerase to telomeres and elongates the telomeres to 2 to 3 times longer than the WT (41). This state corresponds to little if any telomerase action on telomeres, because telomerase is specifically recruited to shorter-than-WT telomeres (42, 43). We performed such preelongation in heterozygous diploids and then selected haploid cells postsporulation that contained a completely WT *CDC13* (and telomerase) locus in a mutant *mrc1*^*AQ*^ *rad9*Δ setting. We did serial streaks and Southern blotting with a telomeric probe to monitor cell growth and telomere length. We analyzed bulk cell cycle progression using cultures prepared as early as possible from freshly isolated spores with this WT telomerase setting of preelongated telomeres and lack of adaptor protein function. FACS analysis revealed a prominent fraction of G_1_ DNA content cells in the population that failed to progress through the cell cycle following synchronized release from an α-factor G_1_ blockade (Fig. 4). Indeed, there was a G_1_ enrichment, even in the asynchronous cells with long telomeres. This result in *mrc1*^*AQ*^ *rad9*Δ cells with WT telomerase present mimicked that seen with genetic deletion of telomerase in the ETI *mrc1*^*AQ*^ *rad9*Δ cells. Therefore, we conclude that lack of telomerase activity on telomeres, rather than simply shorter-than-WT telomeres, causes the observed dependence on adaptor protein function.

**FIG 5:**
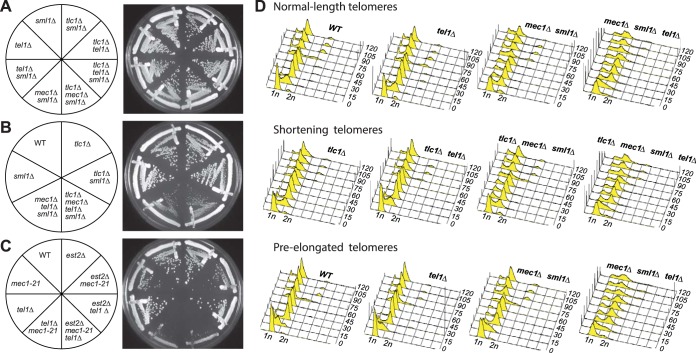
ETI cells do not display growth defects in the absence of Mec1 and Tel1 PIKK kinases. (A) *sml1*Δ, *tel1*Δ, and *mec1Δ sml1*Δ mutations had no effect on bulk colony growth phenotypes of ETI *tlc1*Δ strains (compare left and right halves of the plate). (B) ETI *tlc1*Δ mutation caused no additional growth defects in *mec1 Δtel1Δ sml1*Δ strains (compare left and right halves of the plates). (C) ETI *est2*Δ (as well as *tlc1*Δ and *est2D530A* [not shown]) caused no additional defects in *mec1-21*. All the streaks shown in panels A to C are first-passage cells taken directly from sporulation of diploid strains heterozygous for all the mutatons. (D) The ETI *tlc1*Δ mutation had no effect on the cell cycle profile of the *mec1*Δ and *tel1*Δ strains, regardless of telomere length. FACS analysis of synchronized WT, *tel1*Δ, *mec1Δ sml1*Δ, or *tel1Δ mec1Δ sml1*Δ cells was performed in the context of normal-length telomeres, ETI shortened telomeres, or telomeres lengthened through expression of and then selection against Cdc13-ku70p. Peaks represent DNA content: 1n at start and 2n at full duplication.

We further tested the effect of abrogating the DDR in an ETI setting. Because DDR signaling in S. cerevisiae is initiated at the level of the PIKK kinases Mec1 and Tel1, simultaneously deleting both proteins is another way to eliminate DDR function (17). As mentioned above, Mec1 and Tel1 have a slight and a major role, respectively, in telomere maintenance. Cells lacking Mec1 have bulk telomeres that are only slightly shorter than normal, whereas telomeres in cells lacking Tel1 gradually shorten until they reach a stable length of approximately 150 bp, compared to 250 to 350 bp in WT cells (44). All the *tel1*Δ strains used here were isolated immediately after sporulation of heterozygous diploids and had telomeres that were not significantly shorter than those of TEL1 cells. Mutant *mec1*Δ, *tel1*Δ, and *mec1Δ tel1*Δ strains were created that also contained a *sml1*Δ mutation whenever *mec1*Δ was present in order to suppress the lethality induced by deletion of Mec1 (45). Sml1 is an inhibitor of ribonucleotide reductase (RNR), and deletion of Sml1 is known to elevate nucleotide pools, facilitate DNA replication, and provide resistance to certain forms of DNA damage (45, 46). The bulk viability and colony growth of these strains were compared with and without telomerase activity shortly after the introduction of a *tlc1*Δ mutation to create the ETI strains. The *mec1Δ sml1*Δ and *tel1*Δ strains each grew very well in bulk and showed no change in colony formation phenotypes when an ETI mutation was introduced (Fig. 5A). To our initial surprise, while the combined *mec1Δ tel1Δ sml1* strain was already somewhat sick, the addition of the ETI *tlc1*Δ mutation had no effect on the colony growth formation or cell cycle profile of the strain (Fig. 5B and D). We had shown previously that the *sml1*Δ mutation suppresses ETI phenotypes (35). In order to measure the role of Mec1 function directly, we combined *tel1*Δ with the *mec1-21* allele (47), which allows (somewhat diminished) survival of cells without deletion of Sml1. *mec1-21 tel1*Δ ETI cells had no worse growth than *mec1-21* or *tel1*Δ cells alone, revealing that Mec1 function is not important for viability in ETI cells (Fig. 5C).

### Phenotype rescue by Sml1 deletion is due to relief of RNR inhibition and increase in dNTP pools.

**FIG 6:**
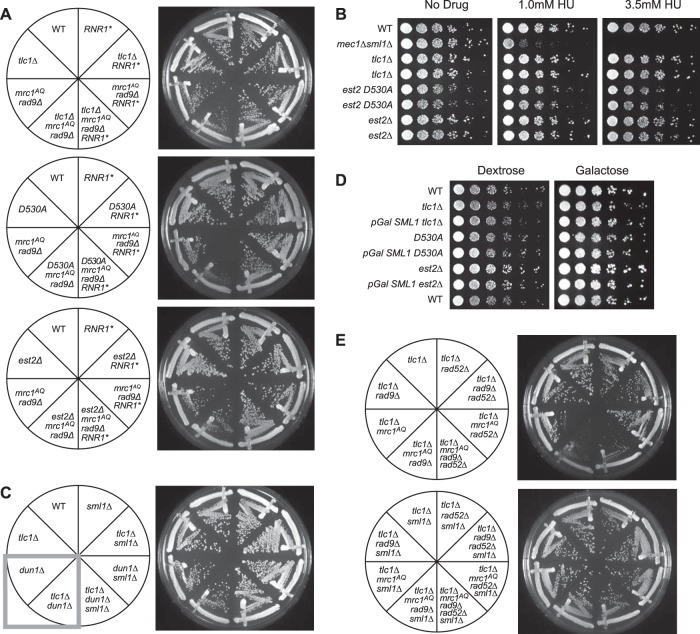
Sml1 rescue is due to increased nucleotide pools. (A) Like *sml1*Δ, RNR1 overexpression (RNR1*) was able to rescue the colony formation defects of ETI *mrc1*^*AQ*^ *rad9*Δ cells for all three ETI mutations. (B) Cells were serially diluted on rich-medium plates containing HU at the indicated concentrations. Despite a strong effect on control *mec1Δ sml1*Δ strains, no significant sensitivity to the drug was observed in ETI cells. Higher doses of the drug (25 mM and 100 mM) showed similar results (data not shown). (C) Dun1, the kinase responsible for phosphorylation and degradation of Sml1, was deleted. (D) Overexpression of Sml1 via a galactose-inducible promoter had no significant effect on growth in ETI strains relative to ETI alone. Frogged plates were incubated for 2 days (dextrose) or 3 days (galactose). (E) Elimination of recombination via Rad52 deletion did not eliminate the ETI *mrc1*^*AQ*^ *rad9*Δ synthetic phenotype or prevent its rescue via Sml1 deletion. All the streaks shown are first-passage cells taken directly from sporulation of diploid strains heterozygous for all the mutations.

The primary known function of Sml1 is the elevation of dNTPs in the cell via inhibition of the RNR enzyme complex (21). However, we wished to investigate whether the alleviation of the ETI *mrc1*^*AQ*^ *rad9*Δ synthetic-sick phenotype might have been due to another unknown function of Sml1. To examine this possibility, we created ETI *mrc1*^*AQ*^ *rad9*Δ strains that overexpressed RNR1, the RNR subunit to which Sml1 specifically binds and that it inhibits (21). Overexpression of RNR1 was able to rescue the ETI *mrc1*^*AQ*^ *rad9*Δ synthetic phenotype to approximately the same extent as in an *sml1*Δ mutant (Fig. 6A). This further supports the interpretation that the rescue of the ETI *mrc1*^*AQ*^ *rad9*Δ synthetic phenotype via Sml1 mutation was due to the relief of inhibition on RNR1 and the consequent increase in the cellular dNTP pools.

### ETI synthetic lethality in the absence of DDR adaptor proteins is not due to sensitivity to small dNTP pools.

Because the DDR is also important for elevating dNTP pools during normal S phase or in response to DNA damage (20, 48), we also tested the possibility that the observed phenotypes arise because ETI cells are especially sensitive to reduced dNTP concentrations that would result from DDR inactivation. We tested ETI strains for reduced bulk population viability compared with WT cells under four different conditions that reduce cellular dNTP concentrations. First, ETI cells were treated with various concentrations of hydroxyurea (HU), a drug that inhibits RNR1, similar to the action of the Sml1 protein (49) (Fig. 6B). Second, we stabilized the Sml1 protein by deleting the gene encoding Dun1, the DDR protein responsible for the phosphorylation and subsequent degradation of Sml1 in response to DDR pathway activation (Fig. 6C), or via use of the *sml1 4SA* mutant that lacks the Dun1 phosphorylation sites and is insensitive to Dun1-mediated degradation (20) (data not shown). Finally, we produced ETI cells with *SML1* under the inducible galactose promoter that greatly overexpress Sml1 when grown on galactose, increasing inhibition of the RNR enzyme complex (Fig. 6D). However, the ETI strains displayed no negative growth effects under any of these conditions. These experiments strongly support the conclusion that bulk growth of ETI single mutants is not sensitive to reduced levels of dNTPs. Therefore, the synthetic lethality induced in ETI cells by Mrc1 and Rad9 deletion cannot be accounted for by the reduction in dNTP pools that results from a defective DDR pathway but is most likely due to the loss of DDR signaling capacity and inability to respond to DNA damage or replication stress.

### Recombination is not required for the synthetic ETI *mrc1*^*AQ*^ *rad9*Δ phenotype or for rescue via Sml1 deletion.

Increased dNTP pools have been shown to repress hyperrecombination in yeast (50). Recombination has also been known to play a role in the survival of cells lacking telomerase. In the absence of telomerase activity, cells may use alternative telomere-lengthening mechanisms involving recombination in order to become “survivor” cells and evade the permanent cell cycle arrest that occurs during senescence (51). We sought to determine if recombination might play a role in the above-mentioned ETI phenotypes. Therefore, we monitored the ETI *mrc1*^*AQ*^ *rad9*Δ synthetic lethality in recombination-deficient cells. Recombination in these strains was prevented by deletion of Rad52, which is known to block recombination in mitotic cells and to prevent the development of survivor cells in the absence of telomerase (51). Deletion of Rad52 had little if any apparent effect on the synthetic-sick phenotype caused by ETI cells in an *mrc1*^*AQ*^ *rad9*Δ genetic background or on its rescue via Sml1 deletion (Fig. 6E). Therefore, recombination does not play a substantial role in the manifestation of these phenotypes.

### The synthetic ETI phenotype with Mrc1 and Rad9 mutation and its rescue via Sml1 deletion are not due to changes in telomere length.

**FIG 7:**
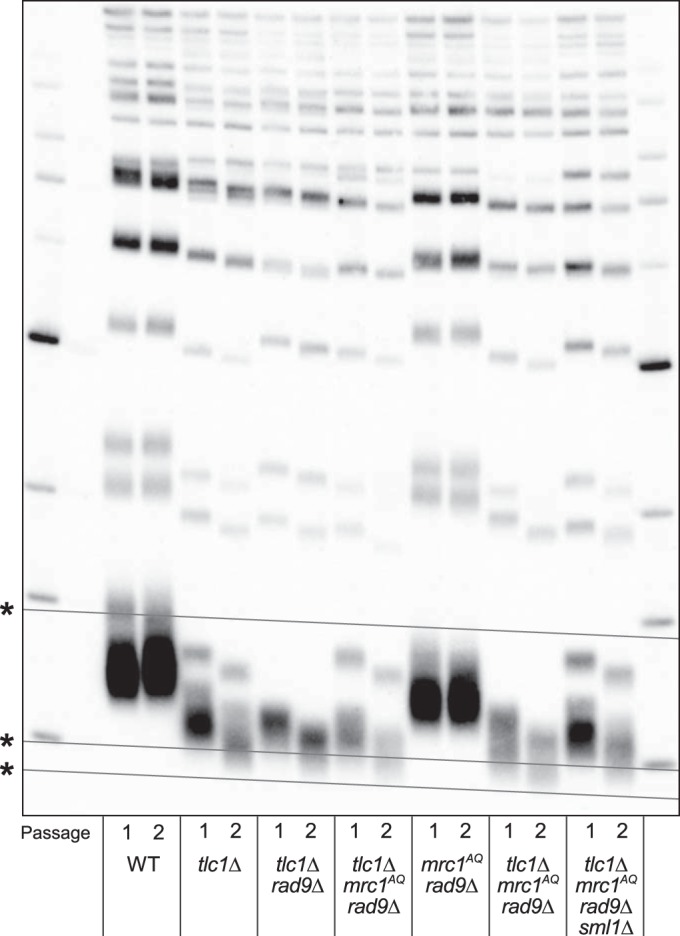
The ETI *mrc1*^*AQ*^ *rad9*Δ synthetic phenotype and *sm1*Δ rescue cannot be explained by changing telomere lengths or accelerated senescence. Shown is Southern blot analysis of terminal XhoI restriction fragments. DNA was probed with an α-^32^P-labeled 5′-(TGTGGG)_4_-3′ Y′-specific probe. The lowest band represents the DNA fragment containing the terminal telomeric repeats. Telomere lengths for the first (1) and second (2) passages after sporulation of a diploid heterozygous strain are represented for all the genotypes. *, grid lines are added to reveal the inherent “smile” in the gel and to allow better comparison of lanes.

A potential simple explanation for the phenotypes described above is that telomeres are drastically shortened in ETI *mrc1*^*AQ*^ *rad9*Δ strains and lengthened by the *sml1*Δ mutation, so the phenotypes observed are the result of premature senescence induced by critically short telomeres. To investigate this, we isolated genomic DNA from the relevant strain genotypes and measured telomere length by performing Southern blot analyses using a telomeric-repeat DNA probe. This technique measures bulk telomere length but is not sensitive enough to detect changes in individual telomeres. We found that while the *tlc1Δ mrc1*^*AQ*^ *rad9*Δ triple mutants did have a shorter bulk telomere length than *tlc1*Δ single mutants, it was apparently only additive with the slightly reduced telomere length seen in *mrc1*^*AQ*^ *rad9*Δ double mutants relative to the WT (Fig. 7). Notably, the telomere length of first-passage (ETI) *tlc1Δ mrc1*^*AQ*^ *rad9*Δ cells (which had low viability and a high fraction of small, noncycling, G_1_ DNA content cells) was no shorter than that of second-passage *tlc1*Δ single mutants, which still grew robustly (Fig. 2 and 3, second passage), with viability similar to that of the WT and no visible signs of senescence. Signs of bulk population senescence typically appear in the third or fourth passage on solid medium (Fig. 3, third passage). Finally, the *tlc1Δ mrc1*^*AQ*^ *rad9Δ sml1*Δ “rescued” mutants showed only very modest telomere length increases at either the first or second passage relative to *tlc1Δ mrc1*^*AQ*^ *rad9*Δ mutants (Fig. 7), despite their greatly improved growth and colony formation phenotypes (Fig. 1A, 2, and 3). Hence, these mild bulk telomere length reductions or increases were not obviously the causative factors for the ETI *mrc1*^*AQ*^ *rad9*Δ synthetic lethality or the *sml1*Δ rescue, respectively. However, while the Southern blots provide a good representation of bulk telomere length distribution, a shorter bulk distribution of telomere length predicts there will be a higher occurrence of critically short telomeres. Therefore, this result does not rule out the possibility of a single critically short telomere (or a few) per cell, which we address below.

### Simultaneously mutating both Mrc1 and Rad9 does not induce synthetic lethality in combination with hypomorphic TLC1 mutants with short telomeres.

**FIG 8:**
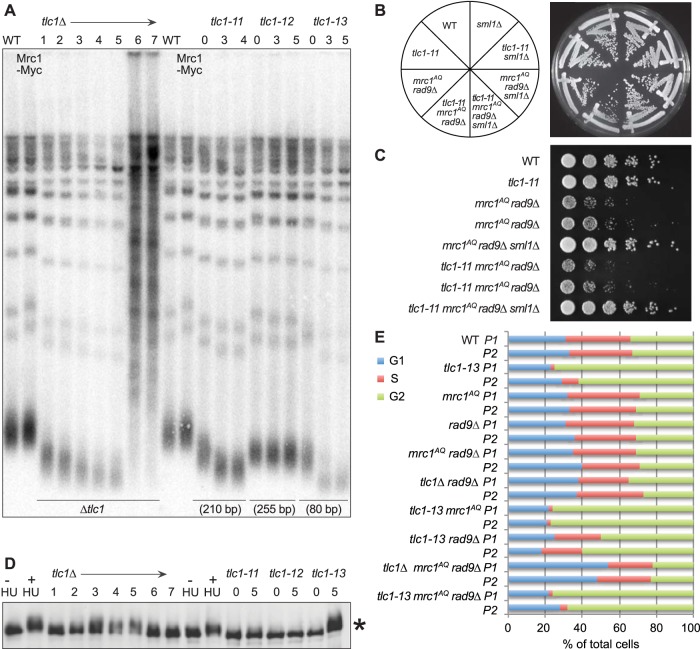
Hypomorphic telomerase does not show a synthetic phenotype with *mrc1*^*AQ*^ *rad9*Δ mutation. (A) Southern blot analysis of terminal XhoI restriction fragments. DNA was probed with an α-^32^P-labeled 5′-(TGTGGG)_4_-3′ Y′ telomere-specific probe on samples from the indicated passages. (B and C) Streaks (B) and serial dilutions (C) of the hypomorphic *TLC1* allele, *tlc1-11*. The *tlc1-11* mutation did not create synthetic lethality with *mrc1*^*AQ*^ *rad9*Δ mutations. In order to ensure shortened telomeres, all the *tlc1-11* haploid strains were obtained by sporulation of a homozygous *tlc1-11/tlc1-11* diploid, which was heterozygous for all the other mutations. (D) MRC1 is phosphorylated as telomeres become critically short. A Western blot of protein extracts was made from the same cultures used in panels A and E and probed with 9E10 anti-myc antibody. The control was WT protein extract treated with 200 mM hydroxyurea for 2 h. *, phosphorylation of Mrc1-myc13 after the indicated passages. (E) Cell cycle phase assignments made from FACS data on samples at 40 (P1) and 60 (P2) generations by plotting the DNA content (CellQuest Pro), followed by curve fitting and quantification of DNA peaks into G_1_, S, and G_2_/M categories (Flow-Jo).

In order to determine if a complete loss of telomerase activity was necessary to induce the synthetic phenotype with the *mrc1*^*AQ*^ *rad9*Δ mutations, these experiments were repeated using hypomorphic TLC1 alleles, *tlc1-11*, *tlc1-12*, and *tlc1-13* (52). Each encodes a telomerase RNA component with a mutation that leads to telomeres that are stable but variously short (Fig. 8A). *tlc1-11* telomeres are 210 bp compared to 350 bp in isogenic WT strains. However, when *tlc1-11* was combined with the *mrc1*^*AQ*^ *rad9*Δ mutations, no synthetic-lethal phenotype was observed (Fig. 8B and C). Furthermore, *tlc1-11* showed no phosphorylation of Mrc1 protein even after 5 passages (100 generations), whereas a second hypomorphic mutant, *tlc1-13*, with even shorter telomeres (80 bp), resulted in phosphorylation of Mrc1, a characteristic of senescent cells, not ETI cells. Phosphorylation of Mrc1 was not seen in *tlc1*Δ cells until they had been without telomerase for three passages (Fig. 8D). As before, ETI combined with loss of DDR adaptor proteins led to an accumulation of cells in G_1_, whereas the hypomorph, *tlc1-13*, paired with the same mutations led to enrichment of cells in G_2_/M after only a single passage (Fig. 8E). This provides further evidence that in ETI cells, it is a lack of telomerase on telomeres, not short telomeres, that leads to dependence on DDR adaptor proteins.

### Decapped and fusogenic telomeres may play a role in ETI *mrc1*^*AQ*^ *rad9*Δ synthetic lethality and *sml1*Δ rescue.

**FIG 9:**
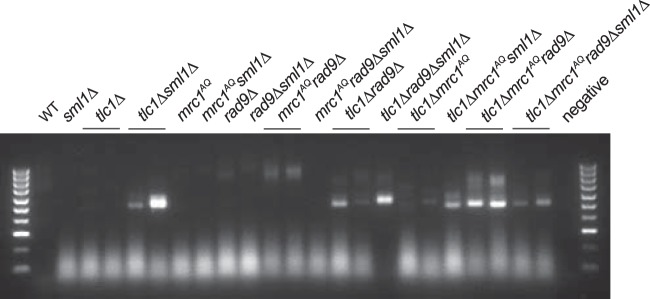
Rescue via Sml1 deletion may be partially due to reduction of deprotected telomeres in strains lacking a functional DNA damage response. A PCR assay was performed to detect deprotected telomeres that fused to an induced double-strand break (HO cut). The intensities of the bands represent the approximate numbers of captured deprotected telomeres. No DNA bands were visible in experiments when either primer was excluded from the PCR. Sml1 deletion had no effect or increased the number of deprotected telomeres detected in strains with a fully or partially functional DDR and reduced the number of deprotected telomeres detected in strains completely lacking a DDR (*mrc1*^*AQ*^ *rad9*Δ).

Telomere deprotection occurs when telomeres become too short to maintain their protective cap of bound proteins. Once this occurs in yeast, telomeres fuse to other critically short telomeres or exposed DNA ends, potentially resulting in broken chromosomes and genomic instability (53, 54). To determine whether the phenotypes studied here were the result of such decapped/fusogenic telomeres, we used a previously published highly sensitive fusogenic telomere capture assay (23). In these experiments, a double-strand break is induced in the left arm of chromosome 7 via HO expression and an integrated HO cut site (55). The cut chromosome acts as a trap for deprotected telomeres, as they are likely to fuse with it. The resulting fusion between chromosome 7 and any deprotected telomere ends can be detected via PCR using primers that hybridize to the subtelomere and to sequences proximal to the HO cut site (23). Surprisingly, the results showed that in strains with a fully or partially functional DDR, addition of the *sml1*Δ mutation either had no effect (e.g., WT or *mrc1*^*AQ*^) or increased (e.g., *tlc1*Δ or *tlc1Δ rad9*) the number of detected deprotected telomeres (Fig. 9). However, in strains completely lacking a functional DDR (e.g., *mrc1*^*AQ*^ *rad9*Δ or *tlc1Δ mrc1*^*AQ*^ *rad9*Δ), addition of the *sml1*Δ mutation noticeably decreased the number of deprotected telomeres detected by the PCR telomere fusion assay. The originally published assay showed that these induced fusions occurred by nonhomologous end joining (NHEJ) and were dependent on the DNA ligase Dnl4 (23). However, deletion of Dnl4 was unable to rescue the ETI *tlc1Δ mrc1*^*AQ*^ *rad9*Δ growth lethality, in contrast to the rescue caused by Sml1 deletion (data not shown). This suggests that, while the *sml1*Δ mutation may play some role in reducing the number of exposed DNA ends in strains lacking a functional DDR, protection from telomere deprotection and telomere fusions is not the sole mechanism through which *sml1*Δ rescues the ETI *tlc1Δ mrc1*^*AQ*^ *rad9*Δ synthetic-sick phenotype. Telomere deprotection and the resulting fusions would be an expected result of telomeric replication stress or fork stalling that would be alleviated by Sml1 deletion. Given the known difficulty of telomere replication (56), we propose that unresolved replication stress in the telomere is the primary source of these phenotypes and that these deprotected telomeres are a symptom of this larger problem.

## DISCUSSION

Here, we have further investigated the phenotypes of cells lacking telomerase that manifest far earlier than previously predicted. It was discovered that ETI cells are completely dependent for viability on functional DDR adaptor proteins and that this DDR requirement is alleviated by deletion of Sml1. These cells were studied when they were still growing comparably to the WT and far from entering telomere shortness-induced senescence that occurs LTI (13, 57). Thus, in ETI cells lacking functional DDR adaptor proteins (ETI *mrc1*^*AQ*^ *rad9*Δ cells), a massive synthetic nonviability was observed that was not accounted for by accelerated telomere shortening or due to inherent sensitivity of ETI cells to reduced dNTP levels. The requirement for Mrc1 (in combination with deletion of Rad9) was specific to Mrc1 activity, as it was not replicated by deletion of Tof1, a partner protein required for Mrc1 localization to the replication fork (25). ETI cells were found to be viable in the absence of functional upstream PIKK sensor kinases (*mec1Δ tel1Δ sml1*Δ), and use of the *mec1-21* allele allowed us to demonstrate directly that Mec1 activity is not essential to the survival of ETI cells. Finally, only a complete loss of telomerase activity (*tlc1*Δ, *est2D530A*, or *est2*Δ) was sufficient to induce the synthetic phenotype with the *mrc1*^*AQ*^ *rad9*Δ mutations, as it could not be replicated using the hypomorphic TLC1 allele, *tlc1-11*. This implies that in the complete absence of a functional DDR, even substantially reduced telomerase activity is able to prevent this synthetic-lethality phenotype.

The rescue of the synthetic phenotype by *sml1*Δ was not caused by bulk telomere lengthening, dependent on recombination, or attributable to a novel function of Sml1 other than its inhibitory effect on RNR1. We found some evidence that the rescue may be partially due to *sml1*Δ reducing the occurrence of deprotected (fusogenic) telomeres in cells lacking a functional DDR. However, this change was not very large, and thus, the presence of other contributing factors seems certain, given the dramatic differences in phenotypes observed between the two genotypes. Because the rescue of the synthetic ETI *mrc1*^*AQ*^ *rad9*Δ phenotype appears to result from the elevated dNTP levels caused by *sml1*Δ, it supports a role of DNA replication stress in this phenotype. Accordingly, all of the data presented here are consistent with the model we previously presented to explain the cell cycle abnormalities and accelerated mother cell aging that also occur in ETI cells (35). In this model, telomerase DNA extension activity acts as an alternative bypass mechanism to stalled or backtracked forks in the telomere. Such a mechanism could include extension of the single-stranded G-rich leading strand revealed by fork backtracking. This is consistent with previous work in fission yeast suggesting that stalled replication forks create a favorable substrate for telomerase, regardless of telomere length (9). It is known that telomeric DNA sequences are extremely difficult regions to replicate due to their repetitive, G-rich sequences and tightly bound protein components (2, 3, 5). Our results indicate that without functional DDR adaptor proteins to respond to and stabilize stalled or backtracked replication forks, the telomerase bypass mechanism becomes substantially more important to ensure proper replication of the telomere. We attribute the synthetic lack of viability to genomic damage resulting from telomeric-fork stalling, as well as fork collapse, which could result in deprotected/fusogenic chromosome ends. In the absence of Sml1, increased dNTP pools facilitate smoother replication through the telomere, preventing fork stalling and fork collapse.

The results presented here suggest that telomerase is required for reasons beyond countering the slow loss of sequence that occurs as cells undergo repeated rounds of DNA replication and cell divisions. Specifically, a loss of telomerase action on telomeres, even when the telomeres are long enough to support growth, burdens cells with genetic dependencies indicative of a reduced ability to resolve telomeric DNA replication stress. Future experiments should focus on determining how telomerase activity on telomeres assists these stalled or collapsed forks. This more continuous need for telomerase to alleviate replication problems in the telomere continues to add to our understanding of telomerase function and telomere maintenance.

## References

[1] Chang M, Luke B, Kraft C, Li Z, Peter M, Lingner J, Rothstein R 2009 Telomerase is essential to alleviate pif1-induced replication stress at telomeres. Genetics 183:779–791. doi:10.1534/genetics.109.107631. 19704012 PMC2778976

[2] Miller KM, Rog O, Cooper JP 2006 Semi-conservative DNA replication through telomeres requires Taz1. Nature 440:824–828. doi:10.1038/nature04638. 16598261

[3] Drosopoulos WC, Kosiyatrakul ST, Yan Z, Calderano SG, Schildkraut CL 2012 Human telomeres replicate using chromosome-specific, rather than universal, replication programs. J Cell Biol 197:253–266. doi:10.1083/jcb.201112083. 22508510 PMC3328383

[4] Meena JK, Cerutti A, Beichler C, Morita Y, Bruhn C, Kumar M, Kraus JM, Speicher MR, Wang Z-Q, Kestler HA, d'Adda di Fagagna F, Günes C, Rudolph KL 2015 Telomerase abrogates aneuploidy-induced telomere replication stress, senescence and cell depletion. EMBO J 34:1371–1384. doi:10.15252/embj.201490070. 25820263 PMC4491997

[5] Sfeir A, Kosiyatrakul ST, Hockemeyer D, MacRae SL, Karlseder J, Schildkraut CL, de Lange T 2009 Mammalian telomeres resemble fragile sites and require TRF1 for efficient replication. Cell 138:90–103. doi:10.1016/j.cell.2009.06.021. 19596237 PMC2723738

[6] Zimmermann M, Kibe T, Kabir S, de Lange T 2014 TRF1 negotiates TTAGGG repeat-associated replication problems by recruiting the BLM helicase and the TPP1/POT1 repressor of ATR signaling. Genes Dev 28:2477–2491. doi:10.1101/gad.251611.114. 25344324 PMC4233241

[7] Buonomo SBC, Wu Y, Ferguson D, de Lange T 2009 Mammalian Rif1 contributes to replication stress survival and homology-directed repair. J Cell Biol 187:385–398. doi:10.1083/jcb.200902039. 19948482 PMC2779251

[8] Lue NF, Chan J, Wright WE, Hurwitz J 2014 The CDC13-STN1-TEN1 complex stimulates Pol α activity by promoting RNA priming and primase-to-polymerase switch. Nat Commun 5:5762. doi:10.1038/ncomms6762. 25503194 PMC4269169

[9] Dehé P-M, Rog O, Ferreira MG, Greenwood J, Cooper JP 2012 Taz1 enforces cell-cycle regulation of telomere synthesis. Mol Cell 46:797–808. doi:10.1016/j.molcel.2012.04.022. 22633956

[10] Chen L-Y, Lingner J 2013 CST for the grand finale of telomere replication. Nucleus 4:277–282. doi:10.4161/nucl.25701. 23851344 PMC3810335

[11] Ray S, Karamysheva Z, Wang L, Shippen DE, Price CM 2002 Interactions between telomerase and primase physically link the telomere and chromosome replication machinery. Mol Cell Biol 22:5859–5868. doi:10.1128/MCB.22.16.5859-5868.2002. 12138196 PMC133977

[12] Vannier J-B, Pavicic-Kaltenbrunner V, Petalcorin MIR, Ding H, Boulton SJ 2012 RTEL1 dismantles T loops and counteracts telomeric G4-DNA to maintain telomere integrity. Cell 149:795–806. doi:10.1016/j.cell.2012.03.030. 22579284

[13] D'Adda di Fagagna F, Reaper PM, Clay-Farrace L, Fiegler H, Carr P, Von Zglinicki T, Saretzki G, Carter NP, Jackson SP 2003 A DNA damage checkpoint response in telomere-initiated senescence. Nature 426:194–198. doi:10.1038/nature02118. 14608368

[14] Dewar JM, Lydall D 2012 Similarities and differences between “uncapped” telomeres and DNA double-strand breaks. Chromosoma 121:117–130. doi:10.1007/s00412-011-0357-2. 22203190

[15] Nautiyal S, DeRisi JL, Blackburn EH 2002 The genome-wide expression response to telomerase deletion in Saccharomyces cerevisiae. Proc Natl Acad Sci U S A 99:9316–9321. doi:10.1073/pnas.142162499. 12084816 PMC123138

[16] Jain D, Cooper JP 2010 Telomeric strategies: means to an end. Annu Rev Genet 44:243–269. doi:10.1146/annurev-genet-102108-134841. 21047259

[17] Melo J, Toczyski D 2002 A unified view of the DNA-damage checkpoint. Curr Opin Cell Biol 14:237–245. doi:10.1016/S0955-0674(02)00312-5. 11891124

[18] Chan SW, Chang J, Prescott J, Blackburn EH 2001 Altering telomere structure allows telomerase to act in yeast lacking ATM kinases. Curr Biol 11:1240–1250. doi:10.1016/S0960-9822(01)00391-8. 11525738

[19] Friedel AM, Pike BL, Gasser SM 2009 ATR/Mec1: coordinating fork stability and repair. Curr Opin Cell Biol 21:237–244. doi:10.1016/j.ceb.2009.01.017. 19230642

[20] Andreson BL, Gupta A, Georgieva BP, Rothstein R 2010 The ribonucleotide reductase inhibitor, Sml1, is sequentially phosphorylated, ubiquitylated and degraded in response to DNA damage. Nucleic Acids Res 38:6490–6501. doi:10.1093/nar/gkq552. 20566477 PMC2965251

[21] Chabes A, Domkin V, Thelander L 1999 Yeast Sml1, a protein inhibitor of ribonucleotide reductase. J Biol Chem 274:36679–36683. doi:10.1074/jbc.274.51.36679. 10593972

[22] Usui T, Ogawa H, Petrini JH 2001 A DNA damage response pathway controlled by Tel1 and the Mre11 complex. Mol Cell 7:1255–1266. doi:10.1016/S1097-2765(01)00270-2. 11430828

[23] Chan SW-L, Blackburn EH 2003 Telomerase and ATM/Tel1p protect telomeres from nonhomologous end joining. Mol Cell 11:1379–1387. doi:10.1016/S1097-2765(03)00174-6. 12769860

[24] Alcasabas AA, Osborn AJ, Bachant J, Hu F, Werler PJH, Bousset K, Furuya K, Diffley JFX, Carr AM, Elledge SJ 2001 Mrc1 transduces signals of DNA replication stress to activate Rad53. Nat Cell Biol 3:958–965. doi:10.1038/ncb1101-958. 11715016

[25] Bando M, Katou Y, Komata M, Tanaka H, Itoh T, Sutani T, Shirahige K 2009 Csm3, Tof1, and Mrc1 form a heterotrimeric mediator complex that associates with DNA replication forks. J Biol Chem 284:34355–34365. doi:10.1074/jbc.M109.065730. 19819872 PMC2797203

[26] Grandin N, Bailly A, Charbonneau M 2005 Activation of Mrc1, a mediator of the replication checkpoint, by telomere erosion. Biol Cell 97:799–814. doi:10.1042/BC20040526. 15760303

[27] Tsolou A, Lydall D 2007 Mrc1 protects uncapped budding yeast telomeres from exonuclease EXO1. DNA Repair 6:1607–1617. doi:10.1016/j.dnarep.2007.05.010. 17618841 PMC2077361

[28] Dolling JA, Boreham DR, Bahen ME, Mitchel RE 2000 Role of RAD9-dependent cell-cycle checkpoints in the adaptive response to ionizing radiation in yeast, Saccharomyces cerevisiae. Int J Radiat Biol 76:1273–1279. doi:10.1080/09553000050134500. 10993638

[29] Al-Moghrabi NM, Al-Sharif IS, Aboussekhra A 2001 The Saccharomyces cerevisiae RAD9 cell cycle checkpoint gene is required for optimal repair of UV-induced pyrimidine dimers in both G(1) and G(2)/M phases of the cell cycle. Nucleic Acids Res 29:2020–2025. doi:10.1093/nar/29.10.2020. 11353070 PMC55462

[30] Vialard JE, Gilbert CS, Green CM, Lowndes NF 1998 The budding yeast Rad9 checkpoint protein is subjected to Mec1/Tel1-dependent hyperphosphorylation and interacts with Rad53 after DNA damage. EMBO J 17:5679–5688. doi:10.1093/emboj/17.19.5679. 9755168 PMC1170896

[31] Lingner J, Hughes TR, Shevchenko A, Mann M, Lundblad V, Cech TR 1997 Reverse transcriptase motifs in the catalytic subunit of telomerase. Science 276:561–567. doi:10.1126/science.276.5312.561. 9110970

[32] Counter CM, Meyerson M, Eaton EN, Weinberg RA 1997 The catalytic subunit of yeast telomerase. Proc Natl Acad Sci U S A 94:9202–9207. doi:10.1073/pnas.94.17.9202. 9256460 PMC23115

[33] Negrini S, Ribaud V, Bianchi A, Shore D 2007 DNA breaks are masked by multiple Rap1 binding in yeast: implications for telomere capping and telomerase regulation. Genes Dev 21:292–302. doi:10.1101/gad.400907. 17289918 PMC1785115

[34] Shore D, Bianchi A 2009 Telomere length regulation: coupling DNA end processing to feedback regulation of telomerase. EMBO J 28:2309–2322. doi:10.1038/emboj.2009.195. 19629031 PMC2722252

[35] Xie Z, Jay KA, Smith DL, Zhang Y, Liu Z, Zheng J, Tian R, Li H, Blackburn EH 2015 Early telomerase inactivation accelerates aging independently of telomere length. Cell 160:928–939. doi:10.1016/j.cell.2015.02.002. 25723167 PMC4496004

[36] Reichard P 1988 Interactions between deoxyribonucleotide and DNA synthesis. Annu Rev Biochem 57:349–374. doi:10.1146/annurev.bi.57.070188.002025. 3052277

[37] Rose MD, Winston F, Heiter P 1990 Methods in yeast genetics. Cold Spring Harbor Laboratory Press, Cold Spring Harbor, NY.

[38] Sambrook J, Russel DW 2001 Molecular cloning, 3rd ed Cold Spring Harbor Laboratory Press, Cold Spring Harbor, NY.

[39] Longtine MS, McKenzie A, Demarini DJ, Shah NG, Wach A, Brachat A, Philippsen P, Pringle JR 1998 Additional modules for versatile and economical PCR-based gene deletion and modification in Saccharomyces cerevisiae. Yeast 14:953–961. 9717241 10.1002/(SICI)1097-0061(199807)14:10<953::AID-YEA293>3.0.CO;2-U

[40] Osborn AJ, Elledge SJ 2003 Mrc1 is a replication fork component whose phosphorylation in response to DNA replication stress activates Rad53. Genes Dev 17:1755–1767. doi:10.1101/gad.1098303. 12865299 PMC196183

[41] Grandin N, Damon C, Charbonneau M 2000 Cdc13 cooperates with the yeast Ku proteins and Stn1 to regulate telomerase recruitment. Mol Cell Biol 20:8397–8408. doi:10.1128/MCB.20.22.8397-8408.2000. 11046137 PMC102147

[42] Bianchi A, Shore D 2007 Increased association of telomerase with short telomeres in yeast. Genes Dev 21:1726–1730. doi:10.1101/gad.438907. 17639079 PMC1920167

[43] Ribeyre C, Shore D 2013 Regulation of telomere addition at DNA double-strand breaks. Chromosoma 122:159–173. doi:10.1007/s00412-013-0404-2. 23504035

[44] Lustig AJ, Petes TD 1986 Identification of yeast mutants with altered telomere structure. Proc Natl Acad Sci U S A 83:1398–1402. doi:10.1073/pnas.83.5.1398. 3513174 PMC323083

[45] Zhao X, Muller EG, Rothstein R 1998 A suppressor of two essential checkpoint genes identifies a novel protein that negatively affects dNTP pools. Mol Cell 2:329–340. doi:10.1016/S1097-2765(00)80277-4. 9774971

[46] Chabes A, Georgieva B, Domkin V, Zhao X, Rothstein R, Thelander L 2003 Survival of DNA damage in yeast directly depends on increased dNTP levels allowed by relaxed feedback inhibition of ribonucleotide reductase. Cell 112:391–401. doi:10.1016/S0092-8674(03)00075-8. 12581528

[47] Sanchez Y, Desany BA, Jones WJ, Liu Q, Wang B, Elledge SJ 1996 Regulation of RAD53 by the ATM-like kinases MEC1 and TEL1 in yeast cell cycle checkpoint pathways. Science 271:357–360. doi:10.1126/science.271.5247.357. 8553072

[48] Hoch NC, Chen ES-W, Buckland R, Wang S-C, Fazio A, Hammet A, Pellicioli A, Chabes A, Tsai M-D, Heierhorst J 2013 Molecular basis of the essential S phase function of the rad53 checkpoint kinase. Mol Cell Biol 33:3202–3213. doi:10.1128/MCB.00474-13. 23754745 PMC3753913

[49] Saban N, Bujak M 2009 Hydroxyurea and hydroxamic acid derivatives as antitumor drugs. Cancer Chemother Pharmacol 64:213–221. doi:10.1007/s00280-009-0991-z. 19350240

[50] Fasullo M, Tsaponina O, Sun M, Chabes A 2010 Elevated dNTP levels suppress hyper-recombination in Saccharomyces cerevisiae S-phase checkpoint mutants. Nucleic Acids Res 38:1195–1203. doi:10.1093/nar/gkp1064. 19965764 PMC2831302

[51] Lundblad V, Blackburn EH 1993 An alternative pathway for yeast telomere maintenance rescues est1-senescence. Cell 73:347–360. doi:10.1016/0092-8674(93)90234-H. 8477448

[52] Lin J, Ly H, Hussain A, Abraham M, Pearl S, Tzfati Y, Parslow TG, Blackburn EH 2004 A universal telomerase RNA core structure includes structured motifs required for binding the telomerase reverse transcriptase protein. Proc Natl Acad Sci U S A 101:14713–14718. doi:10.1073/pnas.0405879101. 15371596 PMC522012

[53] O'Sullivan RJ, Karlseder J 2010 Telomeres: protecting chromosomes against genome instability. Nat Rev Mol Cell Biol 11:171–181. doi:10.1038/nrm2848. 20125188 PMC2842081

[54] Hackett JA, Greider CW 2003 End resection initiates genomic instability in the absence of telomerase. Mol Cell Biol 23:8450–8461. doi:10.1128/MCB.23.23.8450-8461.2003. 14612391 PMC262688

[55] Diede SJ, Gottschling DE 1999 Telomerase-mediated telomere addition in vivo requires DNA primase and DNA polymerases alpha and delta. Cell 99:723–733. doi:10.1016/S0092-8674(00)81670-0. 10619426

[56] Leman AR, Dheekollu J, Deng Z, Lee SW, Das MM, Lieberman PM, Noguchi E 2012 Timeless preserves telomere length by promoting efficient DNA replication through human telomeres. Cell Cycle 11:2337–2347. doi:10.4161/cc.20810. 22672906 PMC3383593

[57] Abdallah P, Luciano P, Runge KW, Lisby M, Géli V, Gilson E, Teixeira MT 2009 A two-step model for senescence triggered by a single critically short telomere. Nat Cell Biol 11:988–993. doi:10.1038/ncb1911. 19597486 PMC4025917

